# Pravastatin and cardiovascular outcomes stratified by baseline eGFR in the lipid-lowering component of ALLHAT 

**DOI:** 10.5414/CN107922

**Published:** 2013-07-02

**Authors:** Mahboob Rahman, Charles Baimbridge, Barry R. Davis, Joshua I. Barzilay, Jan N. Basile, Mario A. Henriquez, Anne Huml, Nelson Kopyt, Gail T. Louis, Sara L. Pressel, Clive Rosendorff, Sithiporn Sastrasinh, Carol Stanford

**Affiliations:** 1 Case Western Reserve University, University Hospitals Case Medical Center, Louis Stokes Cleveland Veterans Affairs Medical Center, Cleveland, OH,; 2 The University of Texas School of Public Health, Houston, TX,; 3 Kaiser Permanente of Georgia, Tucker, GA,; 4 Ralph H. Johnson Veterans Affairs Medical Center, Charleston, SC,; 5 Bronx Nephrology Hypertension, Bronx, NY,; 6 Lehigh Valley Hospital, Allentown, PA,; 7 Tulane University School of Public Health and Tropical Medicine, New Orleans, LA,; 8 Mount Sinai School of Medicine, New York, NY, and the James J. Peters Veterans Affairs Medical Center, Bronx, NY,; 9 Veterans Affairs New Jersey Health Care System, East Orange, NJ,; 10 University of Missouri-Kansas City School of Medicine, Kansas City, MO, USA and; 11 A list of the ALLHAT Collaborative Research Group members has been published previously, in JAMA. 2002; 288: 2981-2997.

**Keywords:** hyperlipidemia, kidney disease, cardiovascular outcomes, chronic renal failure

## Abstract

Background/Aims: The role of statins in preventing cardiovascular outcomes in patients with chronic kidney disease (CKD) is unclear. This paper compares cardiovascular outcomes with pravastatin vs. usual care, stratified by baseline estimated glomerular filtration rate (eGFR). Methods: Post-hoc analyses of a prospective randomized open-label clinical trial; 10,151 participants in the Antihypertensive and Lipid-Lowering Treatment to Prevent Heart Attack Trial (lipid-lowering component) were randomized to pravastatin 40 mg/day or usual care. Mean follow-up was 4.8 years. Results: Through Year 6, total cholesterol declined in pravastatin (–20.7%) and usual-care groups (–11.2%). Use of statin therapy in the pravastatin group was 89.8% (Year 2) and 87.0% (Year 6). Usual-care group statin use increased from 8.2% (Year 2) to 23.5% (Year 6). By primary intention-to-treat analyses, no significant differences were seen between groups for coronary heart disease (CHD), total mortality or combined cardiovascular disease; findings were consistent across eGFR strata. In exploratory “as-treated” analyses (patients actually using pravastatin vs. not using), pravastatin therapy was associated with lower mortality (HR = 0.76 (0.68 – 0.85), p < 0.001) and lower CHD (HR = 0.84 (0.73 – 0.97), p = 0.01), but not combined cardiovascular disease (HR = 0.95 (0.88 – 1.04), p = 0.30). Total cholesterol reduction of 10 mg/dl from baseline to Year 2 was associated with 5% lower CHD risk. Conclusions: In hypertensive patients with moderate dyslipidemia, pravastatin was not superior to usual care in preventing total mortality or CHD independent of baseline eGFR level. However, exploratory “as-treated” analyses suggest improved mortality and CHD risk in participants using pravastatin, and decreased CHD events associated with achieved reduction in total cholesterol. Potential benefit from statin therapy may depend on degree of reduction achieved in total and LDL-cholesterol and adherence to therapy.

**Clinical trials registry: www.Clinicaltrials.gov, NCT00000542**

## Introduction 

It is estimated that more than 20 million Americans have chronic kidney disease (CKD) [1]; it is well established that CKD is associated with a higher cardiovascular disease (CVD) risk [2]. Given the proven efficacy of statins in primary and secondary CVD prevention in the general population, use of statin therapy in patients with CKD appears rational. However, studies evaluating the efficacy of statin therapy in preventing cardiovascular outcomes have typically excluded patients with significantly impaired renal function, though the criteria used for exclusion have varied. In addition, it is thought that “non-traditional” risk factors such as anemia and vascular calcification may contribute to CVD risk in CKD [3]. A recent meta-analysis concluded that statins significantly reduce lipid concentrations and cardiovascular outcomes in patients with pre-dialysis CKD, but failed to improve all-cause mortality [4]. In fact, this paper highlighted the lack of good quality data in this area. Prospective clinical trials in patients with end-stage renal disease (ESRD) have shown no benefits of statin therapy in improving cardiovascular outcomes [5, 6]; however, the combination of ezetimibe 10 mg daily and simvastatin 20 mg daily has been shown to reduce the incidence of major atherosclerotic events in a wide range of patients with advanced CKD [7]. Therefore, whether statin therapy in CKD patients with modest dyslipidemias reduces risk of CVD outcomes remains uncertain. Treatment guidelines from leading authorities also vary; some recommend that cholesterol concentrations be lowered in CKD [8], while others await additional data [9, 10]. 

The lipid-lowering component of the Antihypertensive and Lipid-Lowering Treatment to Prevent Heart Attack Trial (ALLHAT-LLT) examined whether pravastatin compared with usual care reduced mortality in older, moderately hypercholesterolemic, hypertensive participants with at least one additional risk factor for coronary heart disease (CHD) [11]. Previously published results showed no significant difference in all-cause mortality or CHD events (nonfatal myocardial infarction or fatal CHD combined) [11], or progression to ESRD and other clinical renal outcomes between pravastatin and the usual-care group [12]. The modest differential in total cholesterol and LDL-cholesterol between pravastatin and usual care compared with prior statin trials supporting CVD prevention may have contributed to these results based on the traditional intent-to-treat analyses. 

This paper reports post-hoc analyses of the effects of pravastatin therapy compared to usual care on cardiovascular outcomes stratified by baseline estimated glomerular filtration rate (eGFR). We also evaluated associations between achieved reduction of total cholesterol levels and subsequent cardiovascular outcomes, and report exploratory analyses of participants taking statin therapy vs. those who were not in “as-treated” analyses in addition to conventional intent-to-treat analyses. 

## Methods 

ALLHAT adhered to the Declaration of Helsinki and obtained written informed consent. The design and conduct of the ALLHAT-LLT have been reported previously [11, 12]. ALLHAT-LLT was a randomized, non-blinded, large multi-center trial conducted from February 1994 through March 2002 at 513 clinical centers in the United States, Puerto Rico, US Virgin Islands and Canada. The intervention was open-label pravastatin (40 mg/d) vs. usual care. Participants (n = 10,151) were drawn from ALLHAT, a 4-armed antihypertensive trial in which a calcium channel blocker (amlodipine), an angiotensin-converting enzyme-inhibitor (lisinopril), and an α-adrenergic blocking agent (doxazosin) were each compared with a thiazide-like diuretic (chlorthalidone). Eligibility criteria for the ALLHAT-LLT included prior enrollment in ALLHAT (age ≥ 55 years and Stage 1 or 2 hypertension according to the Sixth Joint National Committee for Treatment of Hypertension (JNC-6) with at least 1 additional CHD risk factor) and fasting LDL-cholesterol level of 120 – 189 mg/dl (3.1 – 4.9 mmol/l) for those with no known CHD or 100 – 129 mg/dl (2.6 – 3.3 mmol/l) for those with known CHD. Participants were excluded for: fasting triglyceride levels ≥ 350 mg/dl (3.9 mmol/l), currently prescribed lipid-lowering agents or large doses (≥ 500 mg/day) of nonprescription niacin; significant liver dysfunction (serum alanine aminotransferase (ALT) > 100 IU/l); other contraindications for statin therapy; or known intolerance to statins or secondary cause of hyperlipidemia. Follow-up visits coincided with ALLHAT parent trial visits at 3, 6, 9, and 12 months following randomization and every 4 months thereafter. A fasting lipid profile was obtained for all ALLHAT-LLT participants at LLT baseline, and during follow-up in randomly pre-selected samples of usual-care (5%) and pravastatin (10%) participants. All ALLHAT-LLT participants were advised to follow the National Cholesterol Education Program Step I diet. The usual-care group was treated according to the discretion of their primary care physicians; pravastatin use in the usual-care group was discouraged. 

Serial determinations of serum creatinine and total cholesterol were obtained in a single central laboratory. All baseline data refer to ALLHAT-LLT randomization date. A fasting blood sample was obtained from participants and shipped to a single central laboratory for biochemistry analysis, including measurements of total cholesterol (TC), HDL-C and triglycerides (TG). LDL-C was calculated according to the Friedewald formula: LDL-C = TC – HDL-C – 1/5 TG. Serum creatinine was measured using the Ortho Clinical Diagnostics Vitros Chemistry System (Rochester, NY, USA). The simplified Modification of Diet in Renal Disease (MDRD) study equation was used to estimate GFR according to the formula: (186.3 × serum creatinine^–1.154^ × age in years^–0.203^ × 1.212 (if black) × 0.742 (if female)) [13]. Analyses were repeated using the CKD-Epi equation [14], and the Mayo quadratic [15]. Patients were classified into baseline eGFR (ml/min/1.73 m^2^) strata: mild reduction, normal or increased (≥ 60), and moderate-severe reduction (< 60) [16]. 

The following pre-specified clinical outcomes were assessed: all-cause mortality, a composite of fatal CHD or nonfatal myocardial infarction (MI) (CHD events), combined CVD defined as a composite of the primary outcome, coronary revascularization, hospitalized or otherwise treated angina, stroke, heart failure (fatal, hospitalized or treated without hospitalization) and peripheral arterial disease. Study outcomes were defined in the ALLHAT Manual of Operations, were assessed by site investigators at follow-up visits, and were reported to the ALLHAT Clinical Trials Center (CTC). Medical reviewers from the CTC reviewed all events for concordance with study criteria. More detailed information was collected on a random (10%) subset of CHD and stroke events and was reviewed by the endpoints subcommittee to validate physician diagnoses. For analyses of all-cause mortality, participants who were classified as dead pending confirmation (suspected but unconfirmed deaths), lost to follow-up or refused were classified as withdrawn alive as of their date last known alive. For analyses of CHD and combined CVD, all participants without such events were classified as withdrawn without the event as of their last clinic visit. Data were analyzed according to participants’ randomized treatment assignments regardless of their subsequent medications (intent-to-treat analysis). Baseline characteristics were compared across treatment and baseline eGFR groups using the t-test for continuous covariates and contingency table analyses for categorical data. The Cox proportional hazards model was used to obtain hazard ratios and 95% confidence intervals (CIs) for the clinical outcomes described above. Tests for differences in treatment effects across eGFR groups were performed by calculating the differences in the log likelihoods for models with and without interaction terms. Given the many analyses performed, statistical significance at the 0.05 level should be interpreted with caution. 

“As-treated” analyses were obtained by introducing a “statin treatment” indicator variable as a time-varying covariate into the Cox regression analysis; the resulting adjusted hazard ratios could then be interpreted as to the effect and directionality of treatment crossovers. Due to the relatively high crossover rate, the purpose of these exploratory analyses was to compare participants who were actually taking pravastatin vs. those who were not, in contrast to the traditional intent-to-treat analyses, which compare randomized groups. 

## Results 

**Figure 1.**
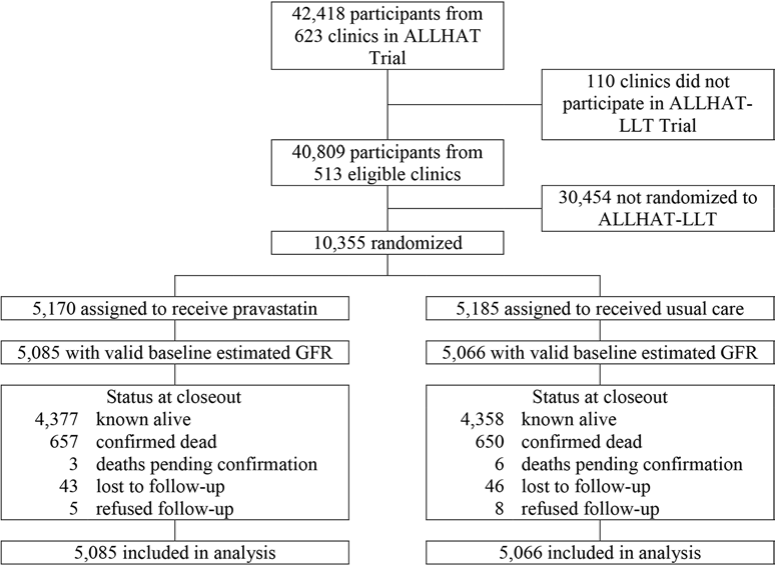
Randomization and follow-up of participants with valid baseline estimated GFR in the Antihypertensive and Lipid-Lowering Treatment to Prevent Heart Attack Trial (ALLHAT).

A description of randomization and follow-up of 10,151 ALLHAT-LLT participants is shown in Figure 1. At baseline, 8,589 participants (84.6%) had mild reduction, normal, or increased eGFR and 1,562 (15.4%) had moderate or severe reduction in eGFR. There were no differences in the baseline characteristics of participants randomized to pravastatin compared with usual care, except for ethnicity (more Black non-Hispanic participants in the pravastatin group, and more white Hispanic participants in the usual-care group) and history of CHD (more in usual care) at baseline in the patients with moderate-to-severe reduction in eGFR (Table 1). 

The mean duration of follow-up was 4.8 years. Adherence to statin therapy in those randomized to pravastatin was 89.8% at Year 2, 86.4% at Year 4, to 87.0% at Year 6. Statin use in participants assigned to usual-care increased from 8.2% at Year 2, to 23.5% by Year 6 [11]. These patterns were consistent across the baseline eGFR strata (Table 2). 

Total cholesterol levels declined by 20.7% in the pravastatin group and 11.2% in the usual-care group with resultant Year 6 total cholesterol levels of 176.2 mg/dl and 196.6 mg/dl, respectively. The changes and differential in total cholesterol between the pravastatin and usual-care groups followed a similar pattern in both eGFR subgroups (Table 2). During the follow-up period, LDL, HDL and triglyceride measurements were available only in a small subset of patients (5% of usual care and 10% of pravastatin). LDL-cholesterol levels declined by 30.2% in the pravastatin group and 15.1% in the usual-care group with resultant Year 6 LDL-cholesterol levels of 103.1 and 121.4 respectively (p < 0.05). There were no statistically significant differences between the pravastatin and usual-care groups with regard to change in HDL-cholesterol or triglyceride between baseline and Year 6. Changes in lipid profiles in eGFR strata were consistent with the overall population, though numbers in individual strata with lipid measures in follow-up were small (Table 2). 

Use of ACE-inhibitors (per antihypertensive treatment trial randomized assignment and open label) was slightly more common in the usual-care group than the pravastatin group at Year 2 (6.2% vs. 4.6% p = 0.002), but not at Year 4 (11.3% vs. 10.9%, p = 0.6) or Year 6 (17.2% vs. 18.6%, p = 0.4). There were no statistically significant differences in systolic blood pressure (SBP) and diastolic blood pressure (DBP) at baseline, 2, 4 or 6 years in the total group (except at 2 years for total), or stratified by baseline eGFR, between the usual-care and pravastatin groups. At 2 years, the mean SBPs were 136.8 and 136.0 in the pravastatin and usual-care groups, respectively (p = 0.03). 

### Clinical outcomes 

**Figure 2.**
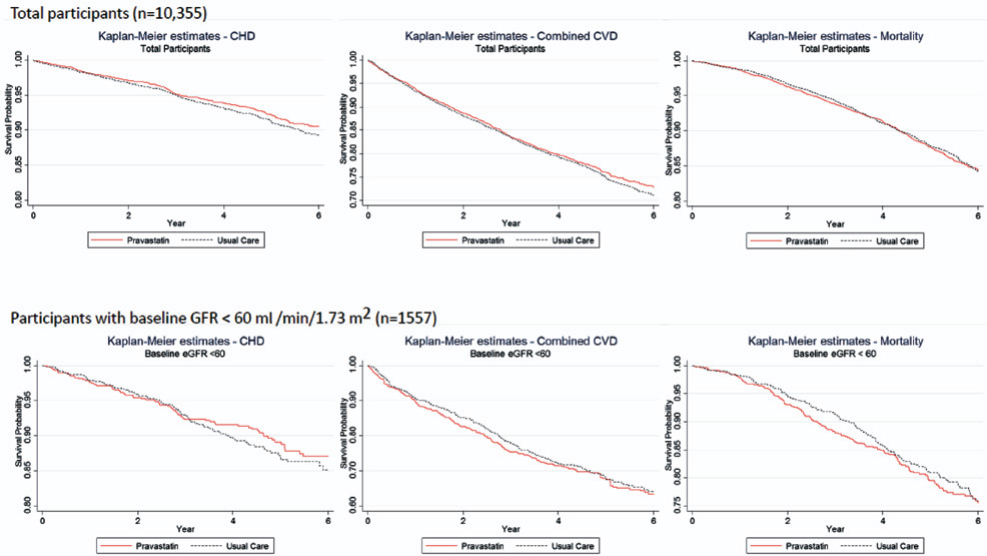
Survival curves for cardiovascular outcomes and mortality – pravastatin versus usual care.

**Figure 3.**
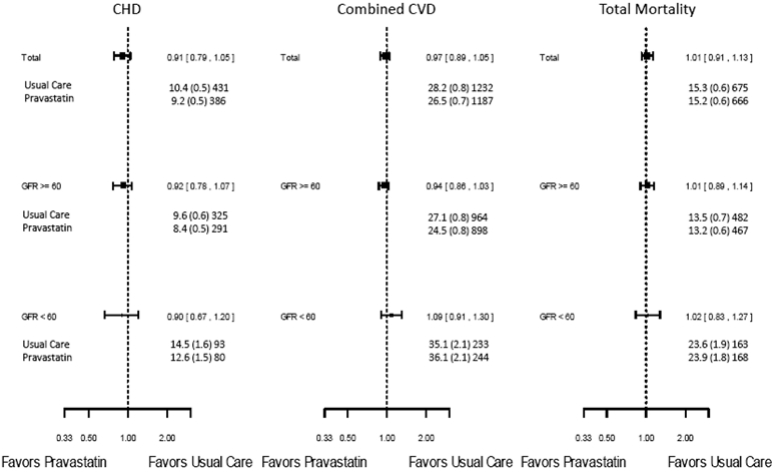
Cardiovascular outcomes and total mortality in the lipid-lowering component of ALLHAT by treatment group and GFR group at baseline (hazard ratios and 95% confidence intervals, 6-year rates per 100, and total events).

There were no statistically significant differences between pravastatin and usual care in 6-year rates of total mortality (15.7 vs. 15.8 per 100, hazard ratio (HR) 1.01, 95% CI 0.91 – 1.13, p = 0.82) or CHD events (9.4 vs. 10.7 per 100, p = 0.11, HR 0.91, 95% CI 0.79 – 1.05, p = 0.20). These overall study findings were similar in both eGFR strata. The p-values for treatment group by eGFR interaction were non-significant for both outcomes (Figure 2, 3). 

There were also no statistically significant differences between pravastatin and usual care in 6-year rates of combined CVD (27.2 vs. 29.0 per 100, HR 0.97, 95% CI 0.89 – 1.05, p = 0.43). There were no significant treatment group differences for combined CVD in the baseline eGFR categories (Figure 3). 

Outcome analyses were repeated with an alternate eGFR stratification (< 45, 45 – 59, and 60+ ml/min). In the eGFR < 45 strata 166 participants were assigned to pravastatin (mean eGFR 37.8 ml/min) and 157 participants were assigned to usual care (mean eGFR 37 ml/min); there were no significant differences between pravastatin and usual care with regard to total mortality (HR = 0.84 (0.57 – 1.22)), CHD (HR = 0.65 (0.35-1.20)) or combined CVD events (HR = 1.24 (0.86 – 1.79)). Analyses were also repeated with alternate equations to estimate GFR (the CKD Epi and the Mayo quadratic equations); results were qualitatively similar in the subgroup of participants with eGFR < 60 using these alternate equations (data in online appendix). 

Among participants with diabetes, there were no differences in treatment group effects across eGFR strata for all-cause mortality or for CHD. For combined CVD, the hazard ratio for diabetic participants with eGFR < 60 (pravastatin/usual care) was 1.42 (95% CI 1.05 – 1.90, p = 0.02), and the hazard ratio for diabetic participants with eGFR ≥ 60 was 0.98 (95% CI 0.85 – 1.02, p = 0.76), p for interaction = 0.03. Hazard ratios (pravastatin/usual care) were consistent across eGFR strata (data not presented). Results were consistent in patients with and without CHD at baseline with regard to total mortality (HR = 1.01 (0.79 – 1.3) vs. HR = 1.02 (0.90 – 1.15)), CHD (HR = 1.02 (0.76 – 1.36) vs. HR = 0.90 (0.77 – 1.05)), or combined CVD (HR = 1.09 (0.92 – 1.29) vs. HR = 0.95 (0.87 – 1.04)). 

Given the relatively high crossover rate, exploratory analyses were performed using “as-treated” analyses comparing participants who were actually taking pravastatin vs. others (as defined above). All-cause mortality (HR = 0.76 (0.68 – 0.85), p = < 0.001) and CHD (HR = 0.84 (0.73 – 0.97), p = 0.01) were significantly lower in the pravastatin group compared to usual care in the as-treated analyses. There were no statistically significant differences between pravastatin and usual care in the as-treated analyses for combined CVD events (Table 3). While in the subgroup of patients with eGFR < 60 there was a significant difference between the pravastatin and usual-care groups for all-cause mortality in the as-treated analyses (HR = 0.78 (0.69 – 0.88), p < 0.001), there was no significant outcome by treatment by GFR interactions. The results for all-cause mortality were consistent when analyses were adjusted for baseline characteristics and time-varying covariates, including achieved cholesterol levels (Table 3). For CHD, the adjusted analyses, which included time-varying cholesterol levels, were not significant for either of the eGFR subgroups or for the combined subgroups. For combined CVD, the adjusted results in the subgroups were consistent with the unadjusted analyses, except that there was a significant eGFR group by treatment interaction (p = 0.03), with the HR for the eGFR group ≥ 60 being 0.95 (0.85 – 1.03, p = 0.17) and for the eGFR group < 60 being 1.18 (0.98 – 1.43, p = 0.08). 

We also evaluated the association between the observed reduction in total cholesterol between baseline and Year 2, with subsequent cardiovascular endpoints in the entire cohort. A 10 mg/dl reduction in total cholesterol was associated with a 5% reduction in risk of CHD events (HR = 0.95 (0.92 – 98), p = 0.001); the interaction term between eGFR group and change in cholesterol was not statistically significant, suggesting that the overall value was the appropriate measure of effect in the subgroups. There was no association with total mortality or combined CVD; these results were consistent across eGFR strata (Table 4). 

## Discussion 

Intent-to-treat analyses of our data showed no beneficial effect of pravastatin therapy over usual care with regard to total mortality or CHD outcomes regardless of baseline eGFR. The total and LDL-cholesterol differential between the randomized groups was relatively small compared to other large lipid-lowering studies, perhaps related to the “drop in” use of statins by participants assigned to usual care. Exploratory “as-treated” analyses, to be interpreted with caution, suggest a benefit for mortality and CHD risk in participants on treatment with pravastatin, and a reduction in CHD events associated with achieved reduction in total cholesterol levels. 

The beneficial effects of statin therapy in both primary and secondary CVD prevention in the general population are well established. However, it is unclear whether the cardiovascular benefits of statin therapy that are observed in the general population extend to the CKD population [17]. This may relate, in part, to the distinct pathophysiology of CVD in CKD with “non-traditional” risk factors such as anemia; accumulation of advanced glycation end-products and calcium-phosphorus abnormalities thought to contribute to CVD risk; and the altered lipid profile associated with CKD [17]. 

To date, few studies have evaluated the effect of statin therapy on cardiovascular outcomes and total mortality in pre-dialysis CKD; most large lipid-lowering studies systematically excluded patients with renal insufficiency. In the Pravastatin Pooling Project, a combined patient-level (n = 4,491 and GFR 30 – 60 ml/min/1.73 m^2^) analysis from 3 randomized trials, pravastatin therapy was associated with a decreased risk for major coronary events compared to placebo [18, 19]. In a recent meta-analyses, fatal (RR = 0.81 (0.73 – 0.90)) and non-fatal cardiovascular events (RR = 0.78 (0.73 – 0.84)) were reduced with statins, but without a significant effect on all-cause mortality (RR = 0.92 (0.82 – 1.03)) [4]. In addition, meta-regression analysis showed that treatment effects did not vary significantly with stage of CKD. In the Prevention of Renal and Vascular End-Stage Disease Intervention Trial (PREVEND IT) in-patients with microalbuminuria, 4 years of treatment with pravastatin did not result in a significant reduction in cardiovascular events (RR = 0.87 (0.49 – 1.57); p = 0.65) [20]. In diabetic [5] and non-diabetic patients [6] on dialysis, treatment with statin therapy has not been shown to improve cardiovascular outcomes. However, results from the Study of Heart and Renal Protection (SHARP) study indicate that a combination of ezetimibe and simvastatin is associated with a reduction in cardiovascular events compared to usual care in patients with CKD [7]. 

The achieved LDL-cholesterol in the patients in the moderate-to-severe reduction in eGFR group in the ALLHAT-LLT (103 mg/dl at Year 2) was similar to the achieved LDL-cholesterol in a similar population in the Pravastatin Pooling Project (103.9 mg/dl at Year 1) [18]. However, the ALLHAT–LLT usual-care group had a decline in LDL-cholesterol resulting in a net difference of 30 mg/dl at Year 2, compared to a difference between pravastatin and placebo of 47 mg/dl at Year 1 in the Pravastatin Pooling Project. The smaller difference in LDL-cholesterol may have contributed to the lack of significant benefit seen with statin therapy in our study. It is also possible that levels of LDL- and total cholesterol achieved in the ALLHAT-LLT are still too high for CKD patients. Whether more aggressive lipid-lowering would result in improved cardiovascular outcomes in these patients remains to be seen. This is supported by our analyses showing that Year 2 reduction in total cholesterol was associated with a lower risk of subsequent CHD. Similarly, the “as-treated” analyses show a reduction in mortality and CHD risk in participants on treatment with pravastatin, supporting a possibility that the observed lack of benefit was a result of crossovers and resulting failure to achieve sufficient difference across treatment groups (the importance of adherence with statin therapy). However, observational analyses of achieved cholesterol reduction, and “as-treated” analyses are not randomized, and may be limited by patient characteristics and other biases. These limitations notwithstanding, these findings support the concept that protocol adherence and achieved reductions in cholesterol levels influence the cardiovascular benefits from statin therapy. 

Our study has several strengths. With more than 1,500 patients with moderately or severely reduced eGFR, this is one of the largest individual studies of statins in patients with renal disease. The 4.8-year mean duration of follow-up is longer than many smaller studies. The methodological rigor of the study with careful event ascertainment and minimal loss to follow-up enhances the credibility of the study. 

There are, however, important limitations to our analyses. Since proteinuria data are not available in ALLHAT participants, we cannot assess the role of proteinuria as a predictor of response to statin therapy. These analyses are post hoc, and therefore should be hypothesis generating, and will await confirmation in other clinical trials. The total and LDL-cholesterol differential between the randomized groups was relatively small compared to other large lipid-lowering studies, perhaps related to statin use by participants assigned to usual care and did not achieve the 30 – 40% reduction in LDL-cholesterol recommended in current lipid guidelines [21]. This may limit the power to detect differences between the two groups. It remains to be seen whether other statins, with greater potency in lipid-lowering than pravastatin, have greater impact on clinical outcomes in this population. For example, recent data suggest that in diabetic patients with CKD and known coronary artery disease, atorvastatin 80 mg was more effective than 10 mg (achieved LDL 79 vs. 99 mg/dl) in reducing risk of CVD [22]. 

Finally, while the as-treated analyses are informative, it is important to note the benefits of randomization are lost in such an analysis, and there may be differences between participants taking pravastatin vs. those who were not that may contribute to differences in outcome. Other factors may have a bearing on the interpretation of our findings. The mean eGFR at baseline in patients in the moderate-severe group (51 ml/min/1.73 m^2^) was higher than in studies that have shown a beneficial effect of statin therapy (most marked in the < 40 ml/min/1.73 m^2^ group in CARE) [23]. However, results in the subset of participants with eGFR < 45 ml/min, albeit a smaller group, did not suggest improved outcomes with pravastatin. 

This paper has important clinical implications. The burden of cardiovascular disease remains high in patients with CKD, and opportunities for intervention to reduce this risk are low. In the context of the results of the SHARP trial, our data support the use of lipid-lowering therapy in patients with CKD to lower cardiovascular risk. 

In summary, this post-hoc analysis of ALLHAT-LLT demonstrates that in hypertensive patients with moderate dyslipidemia, randomization to pravastatin was not superior to usual care in preventing total mortality, or CHD events independent of baseline eGFR level; however, potential benefit from statin therapy may depend on degree of reduction achieved in total and LDL-cholesterol and adherence to therapy. 

## Funding/Support 

This study was supported by contract NO1-HC-35130 with the National Heart, Lung, and Blood Institute. The ALLHAT investigators acknowledge contributions of study medications supplied by Pfizer (amlodipine and doxazosin), AstraZeneca (atenolol and lisinopril) and Bristol-Myers Squibb (pravastatin), and financial support provided by Pfizer. 

## Financial disclosures 

Jan N. Basile has consulted for Boehringer Ingelheim, Daiichi Sankyo, Eli Lilly, Forest Laboratories and Takeda; has received honoraria from Daiichi-Sankyo, Forest Laboratories and Takeda; and has received research support from the National Institutes of Health. 

Barry R. Davis has consulted for Amgen and Takeda. 

Mario A. Henriquez has received honoraria from Astra-Zeneca, Boehringer Ingelheim, Forest Pharmaceuticals and Novartis. 

Nelson Kopyt has received honoraria from Amgen, Novartis and Otsuka. 

Mahboob Rahman has received honoraria and research support from Boehringer Ingelheim. 

Sithiporn Sastrasinh has received honoraria from Abbott Laboratories, AstraZeneca, Baxter International, Bayer Corporation, Boehringer Ingelheim, Bristol-Meyers Squibb, Johnson and Johnson, Merck, Novartis, Pfizer, Roche and Valeant Pharmaceuticals International. 

Charles Baimbridge, Joshua I. Barzilay, Anne Huml, Gail T. Louis, Sara L. Pressel, Clive Rosendorff and Carol Stanford have no financial interests to disclose. 

**Table 1.** Baseline characteristics stratified by estimated GFR* and treatment group.

	Baseline eGFR (ml/min per 1.73 m^2^)					
	Normal/increase/mild decrease (60+)		Moderate/severe (< 60)		Total	
	Pravastatin	Usual care	Pravastatin	Usual care	Pravastatin	Usual care
Number randomized (n, %)	4,302 (50.1)	4,287 (49.9)	783 (50.1)	779 (49.9)	5,085 (50.1)	5,066 (49.9)
Age at lipid randomization – mean (SD)	65.9 (7.3)	65.8 (7.3)	70.8 (7.9)	70.7 (7.9)	66.7 (7.6)	66.6 (7.6)
Ethnicity (n, %)				^a^		
Non-Hispanic						
Non-Black	1,690 (39.3)	1709 (39.9)	405 (51.7)	392 (50.3)^a^	2,095 (41.2)	2,101 (41.5)
Black	1,495 (34.8)	1456 (34.0)	234 (29.9)	209 (26.8)	1,729 (34.0)	1,665 (32.9)
Hispanic						
Non-Black	666 (15.5)	669 (15.6)	89 (11.4)	129 (16.6)	755 (14.9)	798 (15.8)
Black	195 (4.5)	172 (4.0)	14 (1.8)	8 (1.0)	209 (4.1)	180 (3.6)
Other	256 (6.0)	281 (6.6)	41 (5.2)	41 (5.3)	297 (5.8)	322 (6.4)
Women, n (%)	2,030 (47.2)	2052 (47.9)	428 (54.7)	418 (53.7)	2,458 (48.3)	2,470 (48.8)
BMI (kg/m^2^), mean (SD)	30.0 (6.0)	30.0 (6.1)	29.1 (5.7)	29.1 (6.0)	29.8 (5.9)	29.9 (6.1)
Baseline blood pressure (mmHg), mean (SD)						
Systolic	142.6 (17.6)	142.4 (17.6)	145.8 (19.6)	145.8 (20.5)	143.1 (18.0)	142.9 (18.1)
Diastolic	83.0 (10.4)	83.0 (10.3)	82.4 (11.4)	82.1 (11.2)	82.9 (10.5)	82.9 (10.4)
History of CHD at baseline, n (%)	564 (13.1)	611 (14.3)	121 (15.5)	155 (19.9)^a^	685 (13.5)	766 (15.1)^a^
Eligibility risk factors, n (%)^b^						
Current cigarette smoking	1,041 (24.2)	1,028 (24.0)	133 (17.0)	156 (20.0)	1,174 (23.1)	1,184 (23.4)
Atherosclerotic CVD	1,477 (34.3)	1,524 (35.6)	352 (45.0)	357 (45.8)	1,829 (36.0)	1,881 (37.1)
History of MI or stroke	701 (16.3)	706 (16.5)	163 (20.8)	179 (23.0)	864 (17.0)	885 (17.5)
History of coronary revascularization	277 (6.4)	289 (6.7)	64 (8.2)	80 (10.3)	341 (6.7)	369 (7.3)
Other atherosclerotic CVD	827 (19.2)	853 (19.9)	192 (24.5)	205 (26.3)	1,019 (20.0)	1,058 (20.9)
S-T depression on ECG	486 (11.4)	467 (11.0)	98 (12.7)	94 (12.2)	584 (11.6)	561 (11.2)
Type 2 diabetes	1,569 (36.5)	1,509 (35.2)	251 (32.1)	235 (30.2)	1,820 (35.8)	1,744 (34.4)
Low HDL-C	436 (10.1)	451 (10.5)	105 (13.4)	87 (11.2)	541 (10.6)	538 (10.6)
LVH by ECG	816 (19.0)	838 (19.6)	157 (20.1)	152 (19.5)	973 (19.1)	990 (19.5)
LVH by echo	203 (4.8)	197 (4.7)	46 (6.0)	51 (6.6)	249 (5.0)	248 (5.0)
Estimated GFR (ml/min/1.73 m^2^) – mean (SD)*	83.7 (15.7)	83.5 (15.7)	50.8 (8.2)	50.6 (8.4)	78.6 (19.0)	78.5 (19.0)
Lipid baseline lipid profile, mg/dl– mean (SD)						
Total cholesterol^c^	223.1 (27.0)	223.6 (26.3)	226.1 (26.2)	223.7 (28.3)	223.6 (26.9)	223.6 (26.6)
LDL^c^	145.4 (21.4)	145.6 (21.3)	146.5 (21.1)	144.4 (21.4)	145.5 (21.3)	145.4 (21.3)
Fasting triglycerides^d^	148.2 (69.3)	151.1 (69.3)	164.5 (74.3)	164.1 (91.0)	150.6 (70.3)	153.0 (73.1)
Randomized to treatment group, n (%):						
ACE	918 (27.0)	892 (26.5)	161 (26.0)	154 (25.3)	1,079 (26.8)	1,046 (26.3)
CCB	943 (27.7)	921 (27.3)	159 (25.7)	164 (26.9)	1,102 (27.4)	1,085 (27.3)
Diuretic	1541 (45.3)	1556 (46.2)	299 (48.3)	291 (47.8)	1,840 (45.8)	1,847 (46.4)

*Derived from the application of the MDRD study equation based on serum creatinine, age, race and sex. ^a^p < 0.05, comparison between pravastatin and usual care. ^b^For trial eligibility, participants had to have at least 1 other risk factor in addition to hypertension. Thus, the indicated risk factors are not mutually exclusive or exhaustive and may not represent prevalence. ^c^To convert total cholesterol, LDL and HDL to mmol/l, multiply values by 0.0259. ^d^To convert triglycerides to mmol/l, multiply values by 0.0113. BMI = body mass index (calculated as weight in kilograms divided by the square of height in meters); CVD = cardiovascular disease; ECG = electrocardiography; GFR = glomerular filtration rate; HDL-C = high-density lipoprotein cholesterol; LDL = low-density lipoprotein; LVH = left ventricular hypertrophy; MI = myocardial infarction.

**Table 2.** Statin use and lipid levels over the course of the study.

	On statin (study or non-study) n (%)		Total cholesterol mean (SD) n		HDL cholesterol mean (SD) n		LDL cholesterol mean (SD) n		Triglycerides mean (SD) n	
GFR group (ml/min per 1.73 m^2^)	Pravastatin	Usual care	Pravastatin	Usual care	Pravastatin	Usual care	Pravastatin	Usual care	Pravastatin	Usual care
Total										
Baseline	5,085 (100)	0 (0)**	223.6 (26.9) 5,068	223.6 (26.6) 5,056	47.5 (13.4) 5,068	47.4 (13.5) 5,055	145.5 (21.3) 5,063	145.4 (21.3) 5,052	150.6 (70.3) 4,431	153.0 (73.1) 4,439
Year 2	4,069 (89.8)	365 (8.2)**	187.2 (34.7) 3,676	213.8 (34.3)** 3,366	48.9 (14.1) 670	47.3 (15.0) 386	109.7 (30.3) 647	134.6 (30.0)** 369	149.8 (90.8) 498	156.8 (87.9) 298
Year 4	3,055 (86.4)	566 (16.3)**	183.8 (35.1) 2,699	205.8 (36.8)** 2,553	49.5 (14.3) 454	45.2 (12.3)** 300	103.9 (27.6) 437	128.1 (32.2)** 284	142.6 (80.6) 327	174.9 (155.9)* 206
Year 6	943 (87.0)	245 (23.5)**	176.2 (32.9) 829	196.6 (37.1)** 776	47.5 (14.3) 130`	44.5 (15.1) 66	103.1 (28.7) 128	121.4 (35.8)** 64	135.3 (70.6) 85	137.5 (56.6) 44
% Δ Baseline to Year 6			–20.7 (13.3) 824	–11.2 (15.7)** 771	+1.5 (22.4) 130	+3.5 (21.6) 65	–30.2 (19.9) 127	–15.1 (24.9)** 63	+3.9 (54.1) 81	–5.9 (30.9) 39
GFR (≥ 60)										
Baseline	4,302 (100)	0 (0)**	223.1 (27.0) 4,287	223.6 (26.3) 4,278	47.7 (13.3) 4,287	47.5 (13.4) 4,277	145.4 (21.4) 4,284	145.6 (21.3) 4,276	148.2 (69.3) 3,768	151.1 (69.3) 3,777
Year 2	3,474 (90.3)	312 (8.2)**	187.7 (34.9) 3,140	214.4 (34.5)** 2,866	48.8 (13.6) 579	47.6 (15.5) 325	110.7 (30.6) 562	135.0 (30.4)** 310	149.2 (90.2) 429	153.0 (86.7) 251
Year 4	2,623 (86.8)	496 (16.7)**	184.3 (35.3) 2,324	205.7 (36.4)** 2,179	49.8 (14.2) 385	44.7 (12.3)** 262	105.1 (27.4) 373	128.6 (32.1)** 246	138.7 (76.3) 282	181.8 (164.1)** 181
Year 6	792 (87.9)	212 (24.0)**	176.5 (33.4) 693	196.7 (38.0)** 670	46.5 (12.6) 113	45.3 (15.8) 58	104.1 (29.7) 111	119.7 (35.1)* 57	136.3 (71.5) 76	133.3 (53.1) 38
% Δ Baseline to Year 6			–20.3 (13.2) 688	–11.0 (15.9)** 666	+0.4 (20.5) 113	+4.1 (22.7) 57	–29.1 (20.3) 110	–16.3 (23.4)** 56	+4.8 (56.1) 73	–3.1 (31.4) 34
GFR (< 60)										
Baseline	783 (100)	0 (0)**	226.1 (26.2) 781	223.7 (28.3) 778	46.6 (13.8) 781	46.6 (14.1) 778	146.5 (21.1) 779	144.4 (21.4) 776	164.5 (74.3) 663	164.1 (91.0) 662
Year 2	595 (87.1)	53 (7.9)**	184.6 (33.8) 536	210.5 (33.0)** 500	49.5 (16.9) 91	46.0 (12.1) 61	102.7 (27.2) 85	132.2 (27.2)** 59	153.8 (94.7) 69	176.6 (92.5) 47
Year 4	432 (83.9)	70 (13.8)**	180.4 (33.5) 375	206.3 (39.4)** 374	47.9 (15.0) 69	48.4 (12.6) 38	97.0 (28.0) 64	124.7 (33.1)** 38	167.2 (101.2) 45	124.9 (51.4) 25
Year 6	151 (82.5)	33 (21.2)**	174.4 (30.5) 136	196.0 (31.2)** 106	54.4 (21.8) 17	38.1 (5.7) 8	96.5 (21.3) 17	135.0 (41.2)* 7	126.8 (65.4) 9	164.2 (75.3) 6
% Δ Baseline to Year 6			–22.9 (13.3) 136	–12.4 (14.5)** 105	+8.9 (32.1) 17	–1.2 (10.0) 8	–37.2 (15.4) 17	–5.3 (35.7)* 7	–4.5 (30.8) 8	–24.3 (21.5) 5

Comparison between treatment groups: *p < 0.05; **p < 0.001. GFR = glomerular filtration rate.

**Table 3.** Hazard ratios for pravastatin compared to usual care using intention-to-treat, as-treated, and as-treated adjusted for baseline and time varying covariates*.

eGFR group at baseline	Intent to treat			As-treated			As-treated, adjusted*		
	n	HR (95% CI)	p	n	HR (95% CI)	p	n	HR (95% CI)	p
Mortality									
Total	10,145	1.01 (0.91 – 1.13)	0.82	10,145	0.76 (0.68 – 0.85)	< 0.001	10,021	0.73 (0.65 – 0.82)	< 0.001
60+	8,583	1.01 (0.89 – 1.14)	0.88	8,583	0.74 (0.59 – 0.92)	0.007	8,473	0.75 (0.66 – 0.86)	< 0.001
< 60	1,562	1.02 (0.83 – 1.27)	0.83	1,562	0.78 (0.69 – 0.88)	< 0.001	1,548	0.69 (0.54 – 0.87)	0.002
CHD									
Total	10,068	0.91 (0.79 – 1.05)	0.20	10,068	0.84 (0.73 – 0.97)	0.01	9,944	0.88 (0.76 – 1.02)	0.09
60+	8,520	0.92 (0.78 – 1.07)	0.28	8,520	0.85 (0.73 – 1.00)	0.04	8,410	0.89 (0.76 – 1.05)	0.18
< 60	1,548	0.90 (0.67 – 1.20)	0.47	1,548	0.82 (0.61 – 1.11)	0.20	1,534	0.86 (0.62 – 1.18)	0.35
Combined CVD									
Total	10,078	0.97 (0.89 – 1.05)	0.43	10.078	0.95 (0.88 – 1.04)	0.30	9,953	0.98 (0.90 – 1.07)	0.70
60+	8,526	0.94 (0.86 – 1.03)	0.19	8,526	0.93 (0.85 – 1.01)	0.10	8,416	0.94 (0.85 – 1.03)**	0.17
< 60	1,552	1.09 (0.91 – 1.30)	0.36	1,552	1.12 (0.93 – 1.34)	0.23	1,537	1.18 (0.98 – 1.43)**	0.08

CHD = coronary heart disease; CI = confidence interval; CVD = cardiovascular disease; GFR = glomerular filtration rate; HR = hazard ratio. *Lipid trial baseline variables: age, gender, aspirin use, history of coronary heart disease, diabetes, antihypertensive treatment group, body mass index, Black, and time-varying covariates (systolic blood pressure, diastolic blood pressure and total cholesterol); **p for eGFR × treatment group interaction = 0.03.

**Table 4.** Association of 2-year decrease in total cholesterol with subsequent endpoint risk by GFR group in study population, unadjusted.

GFR at baseline	Hazard ratio of 2-year decrease in total cholesterol, per 10 mg/dl cholesterol		
	HR	95% C.I.	p-value for interaction (change in cholesterol × GFR group)
Mortality			
Total	1.00	(0.97 – 1.02)	0.13
60+	0.98	(0.95 – 1.01)	
< 60	1.02	(0.97 – 1.08)	
CHD Events			
Total	0.95	(0.92 – 0.98)*	0.13
60+	0.94	(0.90 – 0.97)*	
< 60	0.99	(0.93 – 1.06)	
Combined CVD			
Total	1.00	(0.98 – 1.02)	0.41
60+	0.99	(0.97 – 1.01)	
< 60	1.01	(0.97 – 1.06)	

*p < 0.05. CHD = coronary heart disease; CI = confidence interval; CVD = cardiovascular disease; GFR = glomerular filtration rate; HR = hazard ratio.

## Supplemental tables

**Supplemental Table 1.** Hazard ratios by eGFR definition: Cox regressions (pravastatin/usual care) using intent-to-treat analyses.

eGFR group at baseline	Equation								
	MDRD			CKD-EPI			Mayo (quadratic)		
	n	HR (95% CI)	p	n	HR (95% CI)	p	n	HR (95% CI)	p
Mortality									
Total	10,145	1.01 (0.91 – 1.13)	0.82	10,145	1.01 (0.91 – 1.13)	0.82	10,145	1.01 (0.91 – 1.13)	0.82
60+	8,583	1.01 (0.89 – 1.14)	0.88	8,244	1.01 (0.89 – 1.15)	0.86	9,293	1.01 (0.90 – 1.14)	0.86
< 60	1,562	1.02 (0.83 – 1.27)	0.83	1,901	1.03 (0.85 – 1.25)	0.79	852	1.02 (0.79 – 1.33)	0.86
CHD									
Total	10,068	0.91 (0.79 – 1.05)	0.20	10,068	0.91 (0.79 – 1.05)	0.20	10,068	0.91 (0.79 – 1.05)	0.20
60+	8,520	0.92 (0.78 – 1.07)	0.28	8,186	0.92 (0.78 – 1.08)	0.30	9,221	0.91 (0.79 – 1.06)	0.23
< 60	1,548	0.90 (0.67 – 1.20)	0.47	1,882	0.91 (0.69 – 1.19)	0.48	847	0.92 (0.63 – 1.34)	0.66
CCVD									
Total	10,078	0.97 (0.89 – 1.05)	0.43	10,078	0.97 (0.89 – 1.05)	0.43	10,078	0.97 (0.89 – 1.05)	0.43
60+	8,526	0.94 (0.86 – 1.03)	0.19	8,190	0.94 (0.85 – 1.03)	0.18	9,230	0.94 (0.86 – 1.03)	0.17
< 60	1,552	1.09 (0.91 – 1.30)	0.36	1,888	1.09 (0.92 – 1.28)	0.32	848	1.20 (0.95 – 1.51)	0.12

CHD = coronary heart disease; CI = confidence interval; CVD = cardiovascular disease; CCVD = combined cardiovascular disease; eGFR = estimated glomerular filtration rate; HR = hazard ratio.

**Supplemental Table 2.** Hazard ratios by eGFR definition: Cox regressions (pravastatin/usual care) using as-treated analyses only.

eGFR group at baseline	Equation								
	MDRD			CKD-EPI			Mayo (quadratic)		
	n	HR (95% CI)	p	n	HR (95% CI)	p	n	HR (95% CI)	p
Mortality									
Total	10,145	0.76 (0.68 – 0.85)	< 0.001	10,145	0.76 (0.68 – 0.85)	< 0.001	10,145	0.76 (0.68 – 0.85)	< 0.001
60+	8,583	0.74 (0.59 – 0.92)	0.007	8,244	0.79 (0.69 – 0.90)	< 0.001	9,293	0.79 (0.70 – 0.89)	< 0.001
< 60	1,562	0.78 (0.69 – 0.88)	< 0.001	1,901	0.75 (0.61 – 0.91)	0.003	852	0.69 (0.53 – 0.90)	0.007
CHD									
Total	10,068	0.84 (0.73 – 0.97)	0.01	10,068	0.84 (0.73 – 0.97)	0.01	10,068	0.84 (0.73 – 0.97)	0.01
60+	8,520	0.85 (0.73 – 1.00)	0.04	8,186	0.87 (0.73 – 1.01)	0.08	9,221	0.86 (0.74 – 1.00)	0.05
< 60	1,548	0.82 (0.61 – 1.11)	0.20	1,882	0.80 (0.61 – 1.05)	0.11	847	0.76 (0.52 – 1.12)	0.17
CCVD									
Total	10,078	0.95 (0.88 – 1.04)	0.30	10,078	0.95 (0.88 – 1.04)	0.30	10,078	0.95 (0.88 – 1.04)	0.30
60+	8,526	0.93 (0.85 – 1.01)	0.10	8,190	0.93 (0.85 – 1.02)	0.12	9,230	0.94 (0.86 – 1.02)	0.15
< 60	1,552	1.12 (0.93 – 1.34)	0.23	1,888	1.09 (0.92 – 1.28)	0.32	848	1.17 (0.93 – 1.48)	0.17

CHD = coronary heart disease; CI = confidence interval; CVD = cardiovascular disease; CCVD = combined cardiovascular disease; eGFR = estimated glomerular filtration rate; HR = hazard ratio.

**Supplemental Table 3.** Hazard ratios comparisons by eGFR definition: multivariate cox regressions (pravastatin/usual care) using as-treated analyses adjusted for baseline and time varying covariates only*.

eGFR group at baseline	Equation								
	MDRD			CKD-EPI			Mayo (quadratic)		
	n	HR (95% CI)	p	n	HR (95% CI)	p	n	HR (95% CI)	p
Mortality									
Total	10,021	0.73 (0.65 – 0.82)	< 0.001	10,021	0.73 (0.65 – 0.82)	< 0.001	10,021	0.73 (0.65 – 0.82)	< 0.001
60+	8,473	0.75 (0.66 – 0.86)	< 0.001	8,142	0.74 (0.64 – 0.86)	< 0.001	9,177	0.76 (0.66 – 0.86)	< 0.001
< 60	1,548	0.69 (0.54 – 0.87)	0.002	1,879	0.71 (0.57 – 0.87)	0.001	844	0.63 (0.47 – 0.85)	0.002
CHD									
Total	9,944	0.88 (0.76 – 1.02)	0.09	9,944	0.88 (0.76 – 1.02)	0.09	9,944	0.88 (0.76 – 1.02)	0.09
60+	8,410	0.89 (0.76 – 1.05)	0.18	8,084	0.90 (0.76 – 1.07)	0.22	9,105	0.90 (0.76 – 1.05)	0.18
< 60	1,534	0.86 (0.62 – 1.18)	0.35	1,860	0.84 (0.62 – 1.12)	0.24	839	0.82 (0.54 – 1.23)	0.34
CCVD									
Total	9,953	0.98 (0.90 – 1.07)	0.70	9,953	0.98 (0.90 – 1.07)	0.70	9,953	0.98 (0.90 – 1.07)	0.70
60+	8,416	0.94 (0.85 – 1.03)**	0.17	8,088	0.93 (0.85 – 1.03)**	0.16	9,113	0.95 (0.87 – 1.04)**	0.26
< 60	1,537	1.18 (0.98 – 1.43)**	0.08	1,865	1.15 (0.97 – 1.37)**	0.11	840	1.28 (1.01 – 1.63)**	0.04

CHD = coronary heart disease; CI = confidence interval; CVD = cardiovascular disease; CCVD = combined cardiovascular disease; eGFR = estimated glomerular filtration rate; HR = hazard ratio. *adjusted with lipid-trial baseline variables: age, gender, aspirin use, history of coronary heart disease, diabetes, antihypertensive treatment group, body-mass index, Black race, and time-varying covariates: systolic blood pressure, diastolic blood pressure and total cholesterol. **p-value for interaction of eGFR group by treatment group = 0.03 for all three eGFR definitions.
